# Comment on ‘Robot-assisted radical cystectomy with intracorporeal urinary diversion: an updated systematic review and meta-analysis of its differential effect on effectiveness and safety’

**DOI:** 10.1097/JS9.0000000000001210

**Published:** 2024-02-16

**Authors:** Xinyi Li, Jiao Huang, Ping Ji

**Affiliations:** a Stomatological Hospital of Chongqing Medical University; b Chongqing Key Laboratory of Oral Diseases and Biomedical Sciences; c Chongqing Municipal Key Laboratory of Oral Biomedical Engineering of Higher Education, Chongqing, People’s Republic of China

*Dear Editor*,

We have read the recent article by Fu *et al*.^1^ on the comparisons of perioperative outcomes between robot-assisted laparoscopic cystectomy with intracorporeal urinary diversion (iRARC) and open radical cystectomy (ORC) in patients with bladder cancer with great interest. Based on a comprehensive analysis of 22 studies involving a total of 7020 patients, the authors demonstrated that iRARC showed superiority in terms of estimated blood loss, blood transfusion rate, length of hospital stay, major complications, and positive surgical margin; however, it was associated with longer operative time and a higher rate of ureteroenteric stricture. We would like to extend our sincere congratulations to the authors for publishing such an outstanding study in the *International Journal of Surgery*. Nevertheless, there are several aspects that require further discussion.

First, conducting a comprehensive literature search is an essential prerequisite for ensuring the reliability of a meta-analysis. However, in this study, the authors failed to include a randomized controlled trial conducted by Dixon *et al*.^2^, which was published in *JAMA Network Open* on 1 June 2023, and reported readmission rates between the two groups. Incorporating this crucial study would mitigate publication bias and enhance the accuracy of this pooled result.

Second, the use of a fixed-effects model for pooled outcomes based solely on a small *I* ^2^ value is not appropriate in meta-analysis. Despite all patients undergoing radical cystectomy, there was significant heterogeneity in terms of preoperative therapy and types of diversion across the included studies. Furthermore, there were notable variations in the conducted time points and countries among these studies. Therefore, it is unjustifiable to assume that each independent study is derived from a sample of the same population. Considering these factors, a random-effects model would be more suitable in this kind of scenario.

Third, in the combined analyses of baseline data, a statistically significant difference was observed in neoadjuvant chemotherapy between the two groups (with a higher proportion in the iRARC group). Neoadjuvant therapy has been demonstrated to induce tissue edema and fibrosis^3^, potentially increasing the surgical complexity for bladder cancer. However, the authors did not further investigate the impact of this within-study variable on perioperative outcomes, such as through subgroup analysis or meta-regression analysis, which may somewhat affect the reliability of the pooled results.

Finally, the author presented a multitude of forest plots showcasing diverse positive and negative analysis results. However, the content within these forest plots is excessively concise, such as lacking annotations for grouping on either side of the invalid line. Enhancing the information density in the forest plot would greatly contribute to enhancing research transparency and study readability.

Overall, we would like to express our sincere appreciation to Fu and colleagues for their dedicated efforts in investigating the effectiveness of iRARC in improving perioperative outcomes after radical cystectomy. They have made significant advancements in this clinically relevant field. However, we would like to raise some concerns that could potentially assist the authors in enhancing the clarity of their findings.

## Ethical approval

No need for this letter to the editor.

## Sources of funding

This study was supported by the National Natural Science Foundation of China (No. 82071115).

## Author contribution

X.L. and J.H.: original draft conception and writing; P.J.: critical revision of the manuscript. All authors reviewed the manuscript.

## Conflicts of interest disclosure

The authors have no conflicts of interest to declare.

## Research registration unique identifying number (UIN)

1. Name of the registry: none.
2. Unique identifying number or registration ID: none.
3. Hyperlink to your specific registration (must be publicly accessible and will be checked): none.

## Guarantor

Ping Ji.

## Provenance and peer review

Commentary, internally reviewed.

## Data availability statement

Not applicable.
